# Understanding the cellular responses to anti‐Abeta antibodies to gain insights into mechanisms of ARIA

**DOI:** 10.1002/alz.085573

**Published:** 2025-01-03

**Authors:** Kate E. Foley, Erica M Weekman, Donna M. Wilcock

**Affiliations:** ^1^ Indiana University School of Medicine/Stark Neurosciences Research Institute, Indianapolis, IN USA; ^2^ Indiana University School of Medicine, Indianapolis, IN USA; ^3^ Indiana University School of Medicine, Stark Neurosciences Research Institute, Department of Neurology, Indianapolis, IN USA

## Abstract

Anti‐amyloid immunotherapy holds great promise for our patients and their families as the first disease‐modifying therapy for the treatment of Alzheimer’s disease (AD) to be approved. Positive clinical trials for lecanamab and donanemab showed significant and rapid lowering of brain amyloid burden and a significant slowing of cognitive decline. Amyloid‐related imaging abnormalities (ARIA) in the form of vasogenic edema (ARIA‐E) and micro ‐ and macro‐ hemorrhages (ARIA‐H) remain the major obstacle to broad use of these agents. Significant cerebrovascular pathology precludes treatment due to enhanced risk of ARIA. In addition, it is known that ApoE4 carriers are at a significantly increased risk of ARIA incidence, with 25‐40% of homozygotes developing ARIA. Understanding the mechanisms underlying ARIA is of critical importance to increase safety and broaden the use of anti‐amyloid immunotherapy.

Using mouse models of amyloid deposition, we have performed systemic and intracranial anti‐beta‐amyloid antibody administration to study the potential mechanisms of both amyloid clearance and cerebrovascular disruptions that lead to ARIA. We have found that microglial activation is present along the vessels that are laden with cerebral amyloid angiopathy (CAA). Furthermore, coincident with microhemorrhage occurrence is increased expression and activity of matrix metalloproteinases (MMPs) MMP9 and MMP3. MMPs are known to degrade basement membranes and tight junction proteins that could lead to disruption of the blood‐brain barrier and, ultimately, edema and hemorrhage. in fact, it has long been understood that MMP9 plays a critical role in the hemorrhagic transformation of ischemic stroke. We have performed single‐cell transcriptomic analysis to examine the glial responses following a single anti‐amyloid immunotherapy and we find significant microglial population shifts and also enhanced signaling from microglia to perivascular macrophages, potentially implicating these cells as a key player in the development of ARIA.

Ultimately, discovering the mechanisms of ARIA will result in the development of safer, next‐generation antibody therapy that has reduced ARIA risk, and also could lead to the identification of adjunct treatments that can be co‐administered with the anti‐amyloid immunotherapy to prevent the occurrence of ARIA in those at risk.

